# Cross-sectional study of prepared foods sold in Indonesian school canteens to inform childhood obesity programs and policies

**DOI:** 10.1017/jns.2025.10068

**Published:** 2026-01-08

**Authors:** Madelyn O. Sijangga, Hastrin Hositanisita, Emma C. Lewis, Hamam Hadi, Mika Matsuzaki, Pamela J. Surkan, Yunhee Kang, Sintha Dewi Purnamasari, Yulinda Kurniasari, Joel Gittelsohn

**Affiliations:** 1 Center for Human Nutrition, Department of International Health, Bloomberg School of Public Health, The Johns Hopkins University, Baltimore, MD, USA; 2 The Department of Nutrition, Faculty of Health Sciences, Alma Ata University, Yogyakarta, Indonesia

**Keywords:** Childhood obesity, Indonesian school canteens, Prepared foods, School food environment, School food vendors

## Abstract

Childhood obesity is an increasing concern in Indonesia, yet little is known about the content and sources of foods offered in Indonesian school food environments. This study aimed to examine the composition and preparation of foods sold in primary school canteens, and to identify potential modifications to address diet-related obesity risk. A cross-sectional survey of canteen vendors (*n* = 10) and structured observations of prepared foods (*n* = 112) sold in canteens were conducted across eight private and public primary schools in Central Java, Indonesia. Foods were categorized by food group, preparation method, and meal type, and associations with factors such as cost, location of sale, and the individual responsible for preparation were analysed using chi-square and *t*-test analyses. Among all prepared foods observed, 73.2% were classified as main meals and 26.8% as desserts, with parents often playing a central role in food preparation. Nearly half (47.3%) of non-beverage items were deep-fried, and the majority of dishes did not align with Indonesian Balanced Nutrition Guidelines. A compositional analysis of each main meal’s ingredients revealed that 29.3% lacked protein and 90.2% did not contain vegetables. Foods that were not deep-fried were priced significantly higher than deep-fried foods (x̄ = Rp.1846 ($0.11) vs Rp.1406 ($0.09); *p* < 0.001). Overall, the majority of prepared foods available to schoolchildren were low in nutritional quality, with limited fruits and vegetables and heavy reliance on frying. These findings highlight the need for strategies that combine parent education on healthy food preparation with economic incentives to increase the accessibility of healthier food options within Indonesian school canteens.

## Introduction

Childhood obesity is a growing public health concern in Indonesia, with one-in-five elementary school-aged children and one-in-seven adolescents estimated to be overweight or obese.^(1,2)^ Indonesia has seen an upward trend in the prevalence of overweight and obesity in children over the past three decades, increasing by 12% between 1996 and 2016.^(3)^ While Indonesia had the 7th highest number of obese children and adolescents globally in 2019, projections suggest that Indonesia will rise to the 4th position by 2030, with an estimated 9 million cases.^(4,5)^

Unhealthy dietary patterns, characterized by high consumption of ultra-processed foods and sugar-sweetened beverages, contribute to this problem.^(1,3,6–11)^ A study in Yogyakarta revealed that children with frequent fruit and vegetable consumption and low junk food consumption were 70% less likely to be obese.^(6)^ Similar findings were observed in other provinces, with the Indonesia Family Life Survey showing that ultra-processed food consumption was associated with increased odds of being overweight.^(7)^ The 2020 Food Security and Nutrition survey found that children in Jakarta, Indonesia’s most populated city, were consuming less nutritious foods compared to 2018, paralleling escalating rates of obesity.^(12)^

These unhealthy dietary patterns are linked to the easy accessibility and affordability of high-calorie, high-processed foods in children’s school food environments. School canteens typically feature one or more vendors contracted to sell food to students.^(13,14)^ A study in East Java found that only 43.5% of schools sold foods that were classified as healthy, and similar findings in other cities revealed widespread availability and consumption of energy-dense foods and sugar-sweetened beverages.^(13–18)^ In Indonesia, children often spend extended hours at school, and obtain a significant portion of their weekly meals from school canteens.^(13,16)^ Most Indonesian children attend school, with more than 24 million children enrolled in elementary school in the 2022–2023 academic year.^(19)^ Elementary school attendance tends to be evenly distributed across location types and gender,^(20)^ and is only slightly higher among children living in a higher socioeconomic status household.^(21)^

Despite approximately 30% of children’s daily calorie intake coming from food and beverages purchased at school canteens, the current school food environment lacks nutrition regulation, with regulations for safety and sanitation rather than nutritional content.^(13,16,22)^ Considering that the majority of school-aged children – regardless of location, gender, or socioeconomic status – derive a significant portion of their daily caloric intake from school canteens, this setting should be a focal point for childhood obesity interventions.

There are multiple opportunities to intervene on nutrition in school food environments to prevent obesity. A majority of successful interventions have been conducted in high-income countries.^(23–28)^ They have involved the direct provision of healthier food items, cost-focused interventions, implementation of nutrition standards and policies, behaviour change initiatives, and marketing strategies.^(23–28)^ However, most of the interventions focused on the internal school food environment were targeted towards federally managed school canteens or aspects such as vending machines and water bottles, and were not conducted in small business-like structures of school vendors, characteristic of Indonesian school canteens.^(23–28)^ In Malaysia, where school canteens share a similar vendor-led structure to Indonesia, a study demonstrated that training vendors on recipe modification and healthier cooking techniques as part of a multi-component intervention led to improved dietary patterns and weight status indicators among primary school students.^(29–31)^ Given that a significant proportion of food items in Malaysian school canteens consist of cooked food, interventions for childhood obesity management in this setting have focused on addressing the healthfulness of prepared foods made by school vendors.^(30–32)^ Similarly, a multi-site case study conducted in Jakarta revealed that the most popular food choices across all evaluated elementary canteens were prepared foods, including rice-based meals and fried noodles.^(15)^ Therefore, specifically directing efforts towards prepared foods could be an effective strategy in a comparable setting.

Although prior studies have examined the infrastructure of Indonesian school canteens, the literature has yet to explore the ingredients and cooking methods of foods sold, particularly as they pertain to obesity risk.^(14,16,33–35)^ Given the need for intervention in this setting, formative research is essential to better understand food offerings and to identify strategies most likely to improve dietary quality in this context. Thus, this study aimed to (1) examine the diversity and characteristics of prepared foods sold in Indonesian school canteens, (2) document the cooking methods used in their preparation, and (3) identify modifiable aspects that could be targeted to enhance nutritional quality and guide tailored strategies for childhood obesity prevention.

## Methods

### Study design and sample

Data were collected in elementary schools located in Bantul (*n* = 6) and Magelang (*n* = 2), Central Java, Indonesia. Bantul is a regency in Yogyakarta, Indonesia, containing 74,085 elementary school students in 364 schools (281 public, 83 private).^(19)^ Magelang is a regency in Central Java, Indonesia, containing 78,565 elementary school students in 603 schools (549 public, 54 private).^(36)^ Data collection occurred between December 2022 and October 2023. The sampling plan was derived from that of a prior study conducted by our team in schools in this area in 2020–2021^(16)^. The resulting study sample consisted of eight elementary schools, selected using a cluster sampling method based on school location (rural or urban) and type of school (private or public). Although this is a small school sample, the research team agreed that data saturation was adequately reached given the abundance and variety of prepared foods observed (*n* = 112), with some schools having multiple vendors (*n* = 10). This study was conducted in accordance with the guidelines of the Declaration of Helsinki. Ethical approval to collect data in these schools was obtained from the Health Research Ethics Committee of Alma Ata University (# KE/AA/XI/10963/EC/2022).

### Survey tools and data collection

We developed a School Canteen and Vendor Observation instrument (Appendix 1) based on input from experts in the field and previously used instruments in similar settings. After iterative refinements and internal testing, the resulting instrument included three main sections: (1) school and vendor information, (2) details about prepared foods sold, and (3) a description of the vendor station setup.

Demographic details in the school and vendor information section included the school’s name, type (public or private), location (urban or rural), and the number of vendors present. After obtaining consent from the headmaster, we surveyed the vendors at each school before each break time to obtain additional information such as vendor location (inside or outside the school), and whether they operated as a mobile or permanent vendor within the school premises. These characteristics were independently observed and recorded by the data collectors. The data collectors were undergraduate students majoring in Nutrition at Universitas Alma Ata. They were comprehensively trained on utilizing the observation form, weighing the foods, and recording the food composition of each food.

Information about prepared foods sold was obtained through surveying canteen vendors, asking about cost, food preparation methods, ingredients, and the food preparation location. Photographs were taken of each food or meal item – both as a complete dish and in its individual components, when appropriate – and attached to the form.

The final section delved into additional details about the vendor’s operations, featuring questions regarding the setup of the vendor station, individuals responsible for preparing the foods, and factors like sanitation or crowdedness. This form was completed for each vendor situated in a school. The information was transcribed in Bahasa Indonesian and translated into English by trained data collectors from Alma Ata University.

### Analysis of food group, preparation method, and meal type

Ingredient lists were categorized into the food groups outlined in Indonesia’s Balanced Nutrition Guidelines and checked against the images of the dish.^(37)^ A food item was categorized as a ‘protein-containing item’ if its ingredient list included an animal or plant protein source, defined by national guidelines as ‘Fish, eggs, poultry, meat, milk, and beans and their processed products (tofu and tempeh).’^(38)^ The presence of the vegetable food group in a dish was indicated if it contained dark green, red and orange, root, stem, or leafy green vegetables.^(39)^ Starchy vegetables were considered only in their unprocessed state; for instance, potatoes sold as French fries were not included. Each of these food group variables was subsequently treated as a dichotomous variable. For example, if a food item had a protein-containing item in its ingredient list, it would be classified as ‘contains protein’; conversely, if it lacked such an ingredient, it would be designated as ‘does not contain protein.’ This classification approach was applied consistently across all food groups. In cases where pictures or ingredient lists were incomplete, the primary researcher and lead author (M.S.) utilized her background knowledge and familiarity with typical ingredients and the composition of the food item to infer its association with the targeted food group, and cross-checked with the data collectors. Items lacking sufficient information for such inferences were excluded from the sample. To ensure the accuracy of these interpretations, a peer debriefing session was conducted with researchers affiliated with Alma Ata University.

Given that Indonesia’s Balanced Nutrition Guidelines recommend limiting consumption of fatty foods to less than 67 grams of oils and fats per day, we prioritized examining deep-frying as a preparation method.^(38)^ This variable was similarly treated as dichotomous, with each item categorized as either ‘deep-fried’ or ‘not deep-fried’. Further subgroup analyses were conducted to compare deep-fried versus non-deep-fried preparation methods for both vegetable-containing and protein-containing food items. Food items that inherently could not be categorized were omitted from the analysis. As a result, the examination of food groups and preparation methods exclusively focused on non-beverage food items. Items with preparation methods listed as ‘drink’ were systematically sorted and excluded from the inferential analyses of prepared foods.

All prepared food items were classified as either ‘main meals’ or desserts based on their preparation methods, ingredients, and background knowledge of how these foods were typically consumed as part of a traditional Indonesian meal pattern. For example, foods that contained components of a traditional Indonesian meal, such as staples like rice or noodles, or side dishes of protein or vegetables, were classified as main meals.

### Data analysis

The translated information from the School Canteen and Vendor Observation instrument was entered into a secure Microsoft Excel Spreadsheet file. Descriptive analysis was then conducted in RStudio (version 4.2.2). To perform a compositional analysis of all food items sold across various vendors in the school canteens, the data file was structured by individual food items, with each data entry representing a singular food item sold.

To understand associations between foods within their specified categories and other independent categorical variables, chi-square tests of independence were conducted. In instances where a group had an observed count of less than 5, Fisher’s exact test was utilized. To understand associations between these foods and independent continuous variables, the normality of these independent variables was checked using the Shapiro–Wilk normality test to determine the suitability of a parametric test. If the test of normality yielded a non-significant *p*-value (*p* > 0.05), Welch’s *t*-test was applied to assess differences among the grouped food items. In cases where the *p*-value of the normality test was below 0.05, the Wilcoxon rank-sum test was employed.

Overall, categorizing each food item based on its relevant characteristics and comparing those that include the targeted food group, or employ a specific preparation method, with those that do not, offers insights into specific and practical opportunities for enhancing the healthiness of these food items and aligning them more closely with Indonesia’s balanced nutrition guidelines.

## Results

### Description of study schools

**Figure 1.**
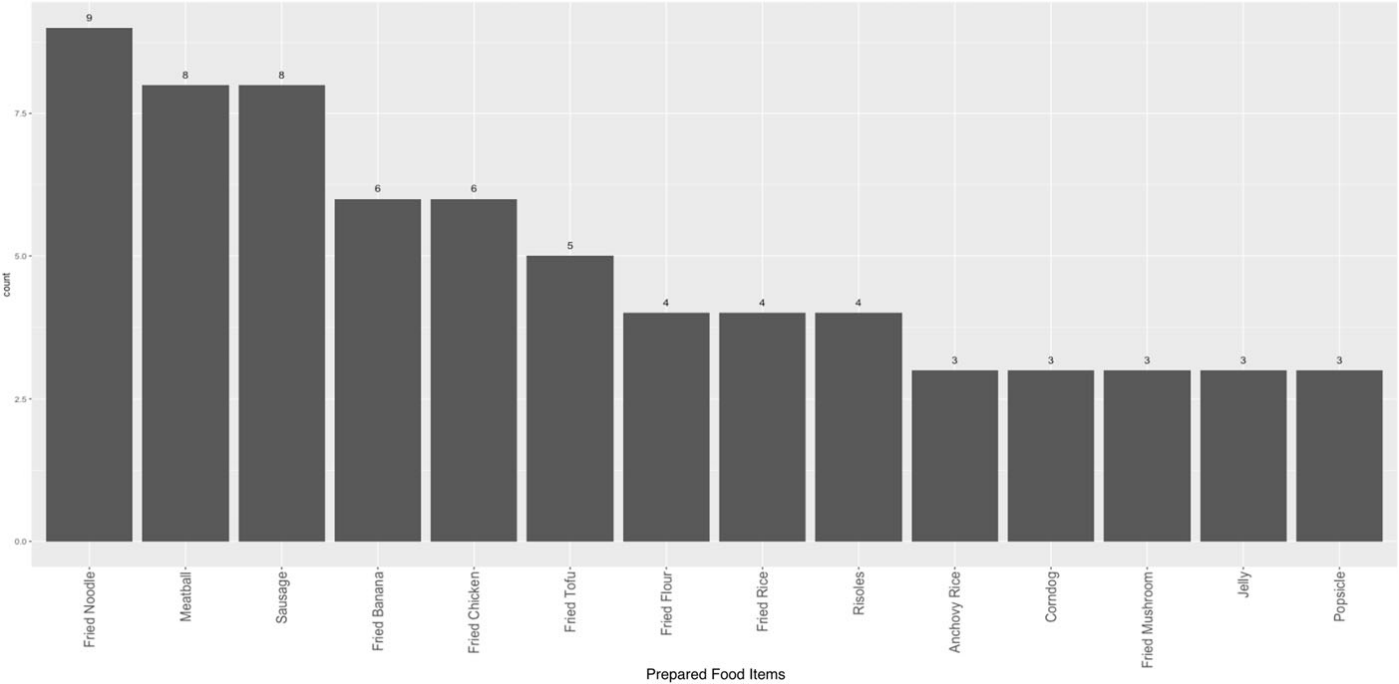
Most commonly sold prepared food items (*n* = 112). Only showing foods with counts > = 3.

Among the eight schools examined, most were public, and a majority were located in urban areas (Table 1). The median number of prepared food items sold per school was 13.5 (range: 10–18). Most schools had just one vendor, and the highest number of vendors observed in a school was two. Fried noodles (*mie goreng*) were the most commonly available dish across all school canteen vendors, comprising 8.0% of prepared foods, followed by meatballs (*bakso*) and sausages (*sosis*) (6.3% each), and fried bananas and fried chicken (5.4% each) (Figure 1).

**Table 1.** Characteristics of the Indonesian elementary schools surveyed in Central Java, Indonesia, from December 2022 to October 2023

	*N* (%)
**School type**	
Private	2.0 (25.0%)
Public	6.0 (75.0%)
**School location**	
Rural	3.0 (37.5%)
Urban	5.0 (62.5%)
**Number of vendors**	
Median (Min, Max)	1.0 (1.0, 2.0)
**Number of food items sold**	
Median (Min, Max)	13.5 (10.0, 18.0)

### Composition of prepared food items sold in Indonesian school canteens

**Figure 2.**
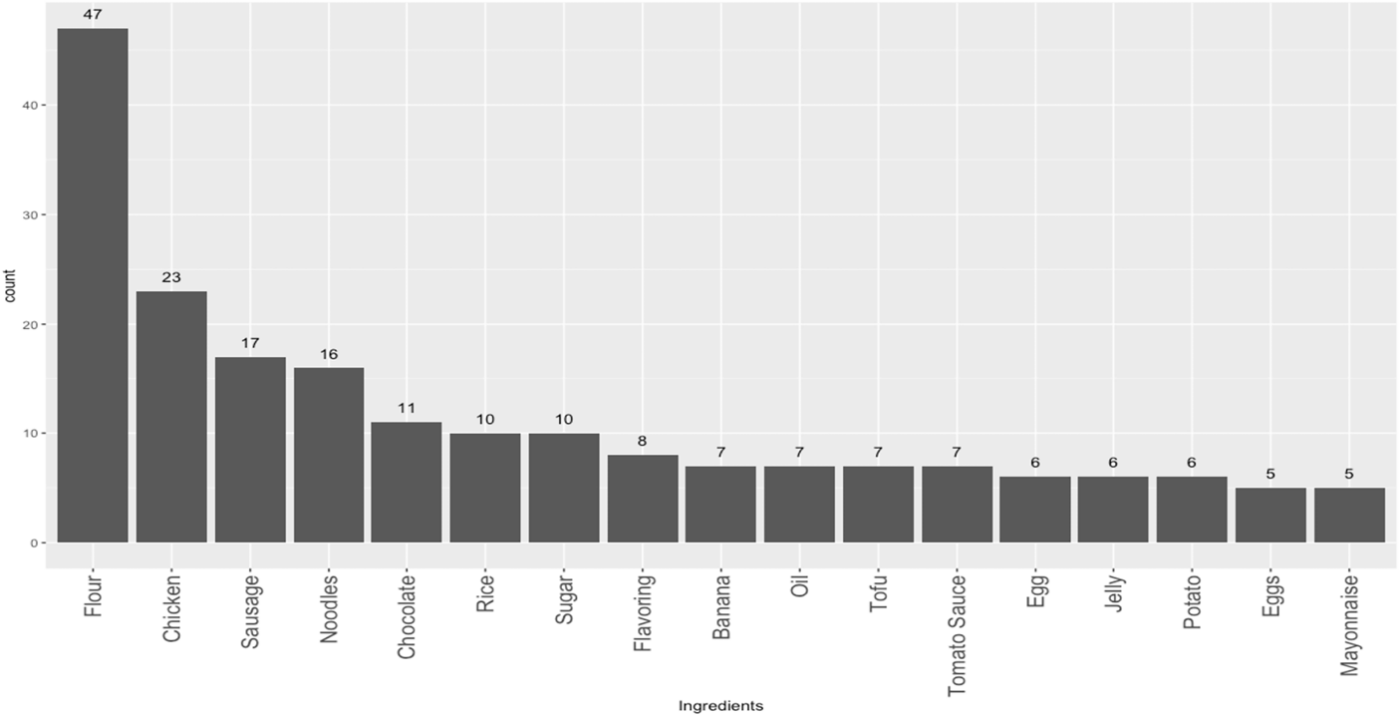
Ingredients utilized across all prepared food items (*n* = 112). Only showing ingredients with counts > = 5.

Out of the 112 prepared food items observed across ten vendors, 73.2% were categorized as main meals and 26.8% were desserts. There were nine beverages sold across all vendors. The average cost of a prepared food item was Rp. 1600 (Indonesian Rupiah), which is equivalent to $0.10 USD (based on the exchange rate of USD $1.00 = Rp. 16,303.5 from 4 February 2025) (Table 2). Most main meal dishes (*n* = 82) lacked protein (Table 3), and 90.2% did not contain any vegetables. Chicken was the most frequently used protein in these dishes, followed by sausage and egg. The most common vegetable used was the mushroom. Desserts and beverage items did not include any proteins or vegetables. Fruits were present in only 7.2% (*n* = 7) of all dishes, with bananas being the most prevalent fruit used. Fresh fruits were sold by just one school food vendor in this survey (watermelon and melon), comprising 28.6% of fruits sold (*n* = 2). The other 71.4% fruits available (*n* = 5) were incorporated into desserts, such as chocolate-covered fried bananas (ex. *pisang molen, pisang cokelat*). Across all dish types, flour was the most common ingredient, appearing in 41.9% of all prepared food items (Figure 2).

**Table 2.** Characteristics of prepared food items sold in Indonesian school canteens in Central Java, Indonesia, from December 2022 to October 2023 (*n* = 112)

**Cost, Indonesian Rupiah (IDR)**	
Mean (SD)	1600.0 (600.0)
Median (Min, Max)	2000.0 (500.0, 3000.0)
**Number of ingredients**	
Mean (SD)	2.3 (1.1)
Median (Min, Max)	2.0 (1.0, 6.0)
**Food type (** ***N*** **(%))**	
Dessert	30.0 (26.8%)
Main	82.0 (73.2%)

**Table 3.** Protein composition of food items sold in Indonesian school canteens in Central Java, Indonesia, from December 2022 to April 2023 (main meals, *n* = 82)

	Protein	*N* (%)
**English**	**Bahasa Indonesia**	
None		24.0 (29.3%)
Chicken	Ayam	23.0 (28.0%)
Sausage	Sosis	14.0 (17.1%)
Soy	Tahu, Tempeh	9.0 (11.0%)
Egg	Telur	5.0 (6.1%)
Fish	Ikan	3.0 (3.7%)
Chicken, soy^a^	Ayam dan Tahu/Tempeh	2.0 (2.4%)
Sausage^b^, egg^a^	Sosis dan Telur	2.0 (2.4%)

a Dish contained both proteins.

b Sausages are typically made from a mix of meats.

### Cooking methods and preparation details

Of the non-beverage food items (*n* = 112), a large portion were reported to be deep fried (47.3%) (Table 4). Other common cooking methods included stir-frying (14.3%) and boiling (13.4%). A subgroup analysis of the fifty-eight main meal food items containing protein revealed that 55% (*n* = 32) were deep-fried. Among the eight vegetable-containing main meal items, only one item (12.5%) was not deep fried, while the remaining 87.5% were in dishes like fried mushrooms (ex*, jamur goreng*), or incorporated as ingredients in other fried items (ex. *tahu isi, bakwan goreng*).

**Table 4.** Summary of the preparation methods of non-beverage food items in Indonesian school canteens in Central Java, Indonesia, from December 2022 to October 2023 (*n* = 112)

	*N* (%)
**Preparation method**	
Deep fried	53.0 (47.3%)
Stir fried	16.0 (14.3%)
Boiled	15.0 (13.4%)
Grilled	9.0 (8.0%)
Baked	6.0 (5.4%)
Frozen	7.0 (6.3%)
Uncooked	3.0 (2.7%)
Steamed	2.0 (1.8%)
Coated	1.0 (0.9%)

A significant portion of all food items was prepared outside of schools’ premises (92.0%) and by parents of the students attending their respective schools (57.1%). These parents were either vendors themselves or dropped off food at the school to be sold. Teachers prepared some food items in a kitchen on school premises (1.8%), while others were prepared off-site by vendors with external relationships to the school (30.4%) or, less frequently, on-site (6.3%). All findings are summarized in Table 5.

**Table 5.** Summary of preparation location and person preparing all food items in Indonesian school canteens in Central Java, Indonesia, from December 2022 to October 2023 (*n* = 112)

	*N* (%)
**Prepared location**	
Off premises not by vendor (by parents)	63.0 (56.3%)
Off premises by vendor	40.0 (35.7%)
On premises by vendor	7.0 (6.3%)
On premises by vendor (by teacher)	2 .0 (1.8%)
**Person responsible for preparing**	
Parents	64.0 (57.1%)
Vendor	34.0 (30.4%)
Parents, Teacher	12.0 (10.7%)
Teacher	2.0 (1.8%)

### Potential factors influencing the healthfulness of food items

There were no significant associations observed between the protein composition of prepared foods and school type, school location, the location of preparation, the individuals responsible for preparation, or cost (Table 6). However, main meal food items containing protein had a higher price (+ Rp. 191 ($0.01)) than foods not containing protein (x̄ = Rp. 1733 ($0.11) vs Rp. 1542 ($0.09); *p* > 0.05), though this difference was not statistically significant.

**Table 6.** Price, school type, school locations, location of preparation, and the individuals responsible for preparation in non-protein-containing foods compared to protein-containing foods (main meals, *n* = 82)

	No protein (*n* = 24)	Protein (*n* = 58)	Wilcoxon rank-sum test/chi-square/Fisher’s exact test *p*-value
**Price in Rp (USD)**	1541.67 ($0.09)	1732.76 ($0.11)	>0.05
**School type**			
Private	5.0	13.0	>0.05
Public	19.0	45.0	
**School location**			
Rural	9.0	22.0	>0.05
Urban	15.0	36.0	
**Preparation location**			
Off school premise	23.0	51.0	>0.05
On school premise	1.0	7.0	
**Individual preparing food**			
Parents	14.0	31.0	>0.05
Teacher	0.0	2.0	
Vendor	10.0	25.0	

A similar pattern emerged for deep-fried foods, which had a significantly lower average cost of Rp. 1406 ($0.09) compared to the average price of Rp. 1846 ($0.11) for non-deep-fried foods (*p* < 0.001) (Table 7). Additionally, a statistically significant association between the preparation method (deep-fried or non-deep-fried) and whether the food items were prepared on or off school premises (*p* < 0.05) was observed. No other statistically significant associations were noted between deep-frying and school type, school location, and who prepared them.

**Table 7.** Price, school type, school locations, location of preparation, and the individuals responsible for preparation in non-deep-fried foods compared to deep-fried-containing foods (non-beverage food items, *n* = 112)

	Not deep fried (*n* = 59)	Deep fried (*n* = 53)	Wilcoxon rank-sum test/chi-square/Fisher’s exact test *p*-value
**Cost in Rp (USD)**	1845.59 ($0.11)	1405.66 ($0.09)	<0.001**
**School type**			
Private	12.0	12.0	>0.05
Public	41.0	47.0	
**School location**			
Rural	22.0	23.0	>0.05
Urban	31.0	45.0	
**Preparation location**			
Off school premise	52.0	56.0	<0.05*
On school premise	1.0	12.0	
**Individual preparing food**			
Parents	33.0	32.0	>0.05
Teacher	0.0	2.0	
Vendor	20.0	34.0	

*Note*: Statistically significant difference at *p*-value: *p < 0.05, **p < 0.001.

When comparing foods containing vegetables to those without, none of these factors, including school type, school location, preparation location and person, and price, showed significant differences (Table 8). However, main meal food items containing vegetables were seen to be lower in price than items without vegetables, although this difference was not statistically significant (x̄ = Rp. 1375 ($0.08) vs Rp. 1709 ($0.10); *p* > 0.05). Analyses of deep-fried and non-deep-fried vegetable-containing dishes were limited by the small sample size of vegetable-containing items (*n* = 8), of which only one was not deep fried.

**Table 8.** Price, school type, school locations, location of preparation, and the individuals responsible for preparation in non-vegetable-containing foods compared to vegetable-containing foods (main meals, *n* = 82)

	No vegetable (*n* = 74)	Vegetable (*n* = 8)	Wilcoxon rank-sum test/Fisher’s exact test *p*-value
**Cost in Rp (USD)**	1709.46 ($0.10)	1375.00 ($0.08)	>0.05
**School type**			
Private	17.0	1.0	>0.05
Public	57.0	7.0	
**School location**			
Rural	27.0	4.0	>0.05
Urban	47.0	4.0	
**Preparation location**			
Off school premise	67.0	7.0	>0.05
On school premise	7.0	1.0	
**Individual preparing food**			
Parents	40.0	5.0	>0.05
Teacher	2.0	0.0	
Vendor	32.0	3.0	

A subgroup analysis comparing the prices of protein-containing dishes found that non-deep-fried items were significantly more expensive than their deep-fried counterparts (x̄ = Rp. 2058 ($0.13) vs Rp. 1469 ($0.09); *p* < 0.001) (Table 9). Similar to the findings for all deep-fried foods, a statistically significant association was observed between the preparation method of protein-containing dishes (deep-fried vs. non-deep-fried) and whether the food was prepared on or off school premises (*p* < 0.05). No significant associations were found with school type, school location, or the individual responsible for food preparation.

**Table 9.** Price, school type, school locations, location of preparation, and the individuals responsible for preparation in protein-containing foods that are non-deep-fried, compared to deep-fried protein-containing foods (*n* = 58)

	Non-deep-fried protein (*n* = 26)	Deep-fried protein (*n* = 32)	Wilcoxon rank-sum test/chi-square/Fisher’s exact test *p*-value
**Cost in Rp (USD)**	2057.692 ($0.13)	1468.750 ($0.09)	<0.001**
**School type**			
Private	6.0	7.0	>0.05
Public	26.0	19.0	
**School location**			
Rural	13.0	9.0	>0.05
Urban	19.0	17.0	
**Preparation location**			
Off school premise	31.0	20.0	<0.05*
On school premise	1.0	6.0	
**Individual preparing food**			
Parents	26.0	19.0	>0.05
Teacher	0.0	2.0	
Vendor	18.0	17.0	

*Note*: Statistically significant difference at *p*-value: *p < 0.05, **p < 0.001.

## Discussion

This is one of the few studies examining the healthfulness of foods sold in primary school canteens in Central Java, Indonesia, and the first to our knowledge to report on the composition and source of prepared foods in this setting. The majority of prepared food items sold in these primary schools did not contain any protein, vegetable, or fruit sources, and were predominantly deep-fried. Among the items that did contain either protein, vegetables, or fruits, the majority were also deep-fried.

The price of these less healthy food options – deep-fried foods, including deep-fried protein-containing items – was significantly lower than their healthier counterparts, namely, non-deep-fried foods and non-deep-fried protein-containing items. Although vegetable-containing items were observed to be lower in price than those without vegetables, this difference was not statistically significant and may be confounded by preparation method, as all but one of the vegetable-containing dishes were deep-fried. These pricing disparities may represent a modifiable factor to support and encourage the purchase of non-deep-fried food options. Overall, our findings confirm that the prepared foods sold in these sampled schools deviate from Indonesia’s Balanced Nutrition Guidelines.^(37)^

The findings from our analysis of prepared foods in school canteens in Central Java presented here align with the observations made in other regions of Indonesia, many of which have similarly found limited diversity in vegetables offered, low availability of fruits, and high availability of deep-fried foods.^(14,40)^ No previous studies have examined protein-containing foods, despite their emphasis in Indonesia’s Balanced Nutrition Guidelines and protein’s important role in influencing energy balance, satiety, and appetite control – all of which are determinants of obesity.^(37,41)^ Only one study, which was conducted in 5 elementary schools in Jakarta, noted that there were fruits and vegetables widely available on 4 of the schools’ canteen menus; however, it was also mentioned that these food options were not typically bought by students.^(15)^ The remaining school, which did not offer foods containing fruits and vegetables in its canteen, attributed its absence to the perception that these foods were unpopular among students.^(15)^ This underscores the potential influence of students’ food preferences and perceptions on the limited availability of healthier food options we observed.

The composition of prepared foods included in the present study aligns with the published literature on the dietary habits of school-aged children in Indonesia.^(10)^ A study conducted in public schools in central Jakarta revealed that a majority of school-aged children did not include vegetables or fruits in their daily diet (71.7% and 57.5%, respectively), and only 3% met the recommended intake for fruits and vegetables.^(10)^ These unhealthy dietary patterns can also be seen in national data, with 93.5% of school-aged children not meeting Ministry of Health guidelines for fruit and vegetable intake, and 41% consuming ‘fatty’ foods more than once a day.^(6,42)^ Cultural food preferences may also influence these trends. In our sample, we observed a mix of traditional Indonesian dishes (e.g. anchovy rice, fried rice) alongside more Westernized items (e.g. sausages, fried chicken, corndogs, and popsicles) (Figure 1). While nutrient analyses of traditional Indonesian staple foods have found that these dishes commonly include nutrient-dense items like green leafy vegetables, research shows they are often consumed in insufficient quantities and inconsistently.^(43)^ Thus, these parallels underscore the importance of considering the availability and appeal of healthier, culturally familiar prepared food options in school canteens to better support dietary habits in children.

Only a handful of studies in this context have conducted a cost analysis on prepared foods sold. One study in Jakarta observed a price difference between less healthy and healthier foods, with reports that deep-fried snacks and sweets had a lower cost than rice-based meals.^(15)^ This aligns with our findings, which show that deep-fried foods had a significantly lower cost compared to non-deep-fried foods. The relationship between healthiness and price has been observed in other settings.^(44)^ A systematic review in high-income countries discovered that fruits and vegetables, followed by protein-based foods, had the highest cost per energy contribution, while foods with added fats and refined starches had the lowest cost.^(44)^ These pricing disparities can be a major barrier to healthier food availability in school settings, especially given that affordability strongly influences students’ food choices. Other studies conducted in Indonesia have found that children’s food choices were primarily determined by the price, with a majority of students’ daily allowances (typically ranging from Rp. 1000 to 5000) spent on canteen foods.^(15,45–48)^ Thus, price-focused strategies could serve as the basis for future interventions to improve the purchasing and consumption of healthier foods in this setting. Fiscal policies, such as implementing taxes on unhealthy items like deep-fried foods and subsidizing healthier options like fresh fruits and vegetables, could help reshape the school food environment. As similar policies are already being discussed for sugar-sweetened beverages in Indonesia, extending them to prepared school foods would be a logical next step.^(49)^

Our finding that parents were primarily responsible for the preparation of most prepared foods sold in primary school canteens in Bantul and Magelang aligns with another study conducted in the same region.^(16)^ In contrast, studies in other regions of Indonesia found that canteen vendors were predominantly teachers, staff members, and relatives of previous vendors.^(14,15)^ This suggests the possibility of regional variations in the individuals responsible for selling school food, and highlights another important factor to consider when devising vendor-focused interventions and conducting future research across Indonesia. While no studies conducted in this environment have formally assessed the understanding of school vendors regarding the healthiness of the food they sell, available literature has explored the nutrition-related knowledge and beliefs of students’ parents. These studies reveal that most parents have limited knowledge of the recommended daily allowances of fruits and vegetables for children, or the adverse effects of unhealthy food consumption.^(10,14)^ Therefore, given that parents were found to prepare the majority of school canteen foods in this setting, developing and implementing clear nutrition guidelines, as well as providing education aligned with Indonesia’s Balanced Nutrition Guidelines to parents, presents a key opportunity to improve the healthfulness of school foods.

Although Indonesia has established national Balanced Nutrition Guidelines, this study highlights a lack of adherence within primary school canteens in Central Java. This may be due to the limited scope of current policies, which have been found to focus more on infrastructure, hygiene, and food safety than on nutritional content.^(14)^ Policy enforcement in this setting would also be essential. A study in this setting found that school canteens were three times more likely to meet health standards when regularly inspected by external officers, such as those from primary health centres or the Indonesian Food and Drug Administration (*Badan Pengawas Obat dan Makanan*).^(16)^ Strengthening both the content and enforcement of school food policies is thus a potential avenue to improve the nutritional quality of foods served in Indonesian school canteens.

In summary, this study highlights key opportunities to improve the healthfulness of prepared foods sold in primary schools in Central Java, Indonesia. Potential strategies include increasing vegetable and fruit availability, incorporating protein, and implementing healthier cooking methods. This study also identified that the primary party responsible for the preparation of these food items was students’ parents, and that price was a key factor influencing the presence of healthier food characteristics. Drawing from these two influential factors of healthfulness we identified, potentially effective interventions could thus incorporate considerations of both pricing and preparation methods. A qualitative study conducted in Malaysia has conceptualized a similar approach for a school-based obesity intervention, implementing training for food vendors to prepare healthier foods and providing subsidies to students to cover the costs of vegetables, fruits, and low-calorie snacks sold in canteens.^(32)^ This intervention resulted in increased availability of healthier food options in their canteens and positive, sustained changes in children’s dietary intake towards these healthier alternatives.^(32)^ Implementing a similar multi-component intervention involving these two identified factors could increase the availability of healthier prepared foods in Indonesian school canteens. However, more in-depth research is needed to understand the existing knowledge and beliefs of school food vendors and parents, as well as students and their purchasing power, to ensure interventions are culturally appropriate and effective in this population.

## Limitations and strengths

There were several limitations worth noting. The small sample size of schools, coupled with the fact that all schools are situated in the same region of Central Java, limits the generalizability of these findings. That said, this study was intended to provide formative findings to inform future research and broader initiatives. While our findings may not reflect the full diversity of school contexts across Indonesia, we believe they provide a useful foundation for broader inquiry. Furthermore, the vendors sampled in this study were primarily internal school vendors; however, outside street vendors also play a large role in shaping schoolchildren’s dietary habits in Indonesia.^(15–17)^ In Malaysia, even when school canteens stop selling unhealthy foods, nearby external vendors may continue to do so.^(32)^ The gram amount of each ingredient of these items was not measured. Consequently, we did not know the exact gram amount of ingredients such as protein or vegetables, and there could potentially be a small amount of these key ingredients served in each dish. This study did not observe students’ purchasing behaviours, limiting insights into which prepared foods and beverages are most commonly selected and how students combine these items to form typical meals. Future research should examine purchasing patterns to better understand the potential impact of canteen offerings on children’s typical dietary intake and its implications for childhood obesity. Nevertheless, a strength of this study is its mixed-methods approach, incorporating both observations and surveys conducted with vendors. This approach offered a comprehensive and objective perspective compared to prior studies typically focused on observational data collection. The observed diversity and abundance of foods surveyed in this sample also provide a basis for understanding the range of foods typically available in primary school canteens in Central Java, Indonesia.

## Conclusion

In conclusion, this study identified that the majority of foods sold in primary schools in Central Java, Indonesia lack vegetables, fruits, and protein sources and are deep-fried. We also observed that price may influence food choices, as unhealthier options – particularly deep-fried items – were significantly less expensive than healthier alternatives. Given that the students’ parents were identified as the primary food preparers, targeted nutrition education aimed at parents may be an effective strategy to improve food preparation and promote healthier cooking methods. Additionally, interventions that have successfully lowered the cost of healthier foods and encouraged healthier preparation techniques in comparable contexts provide a valuable framework for future efforts. These findings offer insights to inform and guide the development of childhood obesity prevention programs and policies within Indonesian school environments.

## Supporting information

Sijangga et al. supplementary material 1 Sijangga et al. supplementary material

Sijangga et al. supplementary material 2 Sijangga et al. supplementary material

## Supplementary material

The supplementary material for this article can be found at https://doi.org/10.1017/jns.2025.10068
