# Supplementary material for: Cross-sectional study of prepared foods sold in Indonesian school canteens to inform childhood obesity programs and policies
Source: J Nutr Sci. 2026 Jan 8;15:e7. doi: 10.1017/jns.2025.10068 (PMC12800532; doi:10.1017/jns.2025.10068)
Supplement: Sijangga et al. supplementary material 1 — Sijangga et al. supplementary material [file S2048679025100682sup001.docx]

**Appendix 1: School Canteen and Vendor Observation Form (Baseline)**
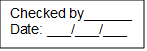


**Time Data Collection Started:** ______:_______ AM/PM

Data Collector Name: _________________________________________ Date: _____/_____/_____ Day of the week:____________________

School Name: _________________________________________

School ID Number: _____________________________________________
Type of School (circle one): Public or Private

School Location (circle one): Rural or Urban or Suburban

Grades Served: ____________________________

Number of Vendors: __________________________

Does the School have a “No Outside Vendors” Policy? (circle one): Yes or No

**Vendor Information**

1. Kiosk Name: __________________________________
2. Indoor Vendor? Yes or No
3. Outdoor Vendor? Yes or No
4. Mobile Vendor? Yes or No
5. Permanent Vendor? Yes or No

**Prepared Foods Sold at Vendor**

| **Food/Beverage** | **Serving size** | **Cost per Serving** | **Cooking/Food Preparation Method** | **Composition of Food/Meal** | **Where is it prepared?**   1. **Off premises NOT by vendor** 2. **Off premises by vendor** 3. **On premises by vendor** | **Comments**  **(Most expensive food, least expensive, most purchased food, etc.)** |
| --- | --- | --- | --- | --- | --- | --- |
|  |  |  |  |  |  |  |
|  |  |  |  |  |  |  |
|  |  |  |  |  |  |  |
|  |  |  |  |  |  |  |
|  |  |  |  |  |  |  |
|  |  |  |  |  |  |  |
|  |  |  |  |  |  |  |

**Packaged Foods Sold at Vendor**

Number of pre-packaged **BEVERAGES** sold at vendor (circle one): 1-5 6-15 16-25 26+

Number of pre-packaged **CANDIES** sold at vendor (circle one): 1-5 6-15 16-25 26+

Number of pre-packaged **SNACKS** sold at vendor (circle one): 1-5 6-15 16-25 26+

**Other Information**

Describe the vendor station (ex. A table, off the back of a motorcycle)

____________________________________________________________________________________________________________________________________________________________________________________________________________________________________________________________________________________________________________________________________________________________________________________________________________________________________________________________________

Describe any posted signage at the vendor station

____________________________________________________________________________________________________________________________________________________________________________________________________________________________________________________________________________________________________________________________________________________________________________________________________________________________________________________________________

Final Comments (ex. sanitation, how busy it is, anything not covered above, etc.)

____________________________________________________________________________________________________________________________________________________________________________________________________________________________________________________________________________________________________________________________________________________________________________________________________________________________________________________________________
